# Clinicopathological and prognostic significance of SDC1 overexpression in breast cancer

**DOI:** 10.18632/oncotarget.22820

**Published:** 2017-11-30

**Authors:** Xiangrong Cui, Xuan Jing, Qin Yi, Chunlan Long, Jie Tian, Jing Zhu

**Affiliations:** ^1^ Pediatric Research Institute, Children’s Hospital of Chongqing Medical University, Ministry of Education Key Laboratory of Child Development and Disorders, Chongqing 400014, China; ^2^ China International Science and Technology Cooperation Base of Child Development and Critical Disorders, Chongqing 400014, China; ^3^ Chongqing Key Laboratory of Pediatrics, Chongqing 400014, China; ^4^ Cardiovascular Department (Internal Medicine), Children’s Hospital of Chongqing Medical University, Chongqing 400014, China; ^5^ Clinical Laboratory, Shanxi Province People’s Hospital, Taiyuan 030000, China

**Keywords:** syndecan-1, breast cancer, bioinformatic analyses, prognosis

## Abstract

**Background:**

Breast cancer is the leading cause of cancer death among global women, and its early diagnosis and treatment are very urgent. Syndecan-1 (SDC1) is a heparin sulfate proteoglycan, which has been linked with the prognosis and treatment response in a various tumor type. To investigate whether SDC1 can serve as a prognostic indictor in breast cancer, bioinformatic analyses were performed in the present study.

**Methods:**

SDC1 expression was assessed using Oncomine analysis. Kaplan-Meier Plotter and bc-GenExMiner were performed to identify the prognostic roles of SDC1 in breast cancer. COSMIC analysis and cBioPortal database were performed to analysis the mutations of SDC1. The heat map and methylation status of SDC1 were identified by performing the UCSC.

**Results:**

We found that SDC1 was more frequently overexpressed in breast cancer than their normal tissues and its expression might be negatively related with some CpG sites. Meanwhile, pooled data suggested that SDC1 mRNA expression is associated worse prognosis of breast cancer. Following data mining in multiple big databases confirmed a positive correlation between SDC1 mRNA expression and PLAU mRNA expression in breast cancer tissues. In addition, high SDC1 expression is associated with increased risked of age, nodal, HER2 and higher SBR grade status.

**Conclusion:**

Our findings suggest that overexpressed SDC1 was identified in breast cancer than in matched normal tissues and is associated with methylation status of SDC1 promoter. Additionally, SDC1 is positively associated with PLAU and might act as a potential prognostic indicator for breast cancer.

## INTRODUCTION

Breast cancer is the most frequently diagnosed female tumor type, and its incidence continues to rise [1, 2]. Despite the development in early clinical diagnosis and treatment strategies, breast cancer remains a significant global burden [3, 4]. Detection of breast cancer at an early stage is a critical step for successful treatment and improvement of prognosis. Therefore, to identify sensitive and specific biomarkers for prognosis of breast cancer patients has important clinical implications.

Syndecan family is a new found evolutionarily conserved transmembrane proteoglycan which can regulate cell proliferation, migration, adhesion, angiogenesis, and modulate cell-cell and cell-matrix interactions during wound reparation [5, 6]. Syndecan-1 (SDC1) is an important member of syndecan family, which expression has been associated with prognosis and treatment response in a various tumor types, including solid tumors and hematolymphoid malignancies [7–14]. SDC1 has also been demonstrated to be enhance endometrial cancer invasion by modulating matrix metalloproteinase-9 (MMP-9) expression through nuclear factor kappaB (NF-κb) [15]. In addition, the proliferation and invasion of glioma cell has been attenuated through knockdown SDC1 [5]. Moreover, syndecan-1 has been identified at high levels in a significant percentage of breast carcinomas and related to an aggressive phenotype and poor clinical behavior [16]. Together, SDC1 may not only act as a potential therapeutic target, but also as a novel prognostic biomarker in cancer. However, the role of SDC1 in breast cancer and the underlying molecular mechanism responsible for its involvement in the generation and development of tumor are still unclear. The clinicopathological and prognostic value of SDC1 expression patterns in breast patients remains controversial [17, 18].

In this study, we evaluated the significance of SDC1 mRNA in human breast cancer using Oncomine microarray datasets. We further analyzed the relationship between SDC1 expression and clinicopathologic parameters including prognostic significance, and explored the biological function and molecular mechanism of SDC1 in breast cancer patients by pooling all currently available data.

## RESULTS

### SDC1 transcript expression and methylation status in human breast cancer.

**Figure 1:**
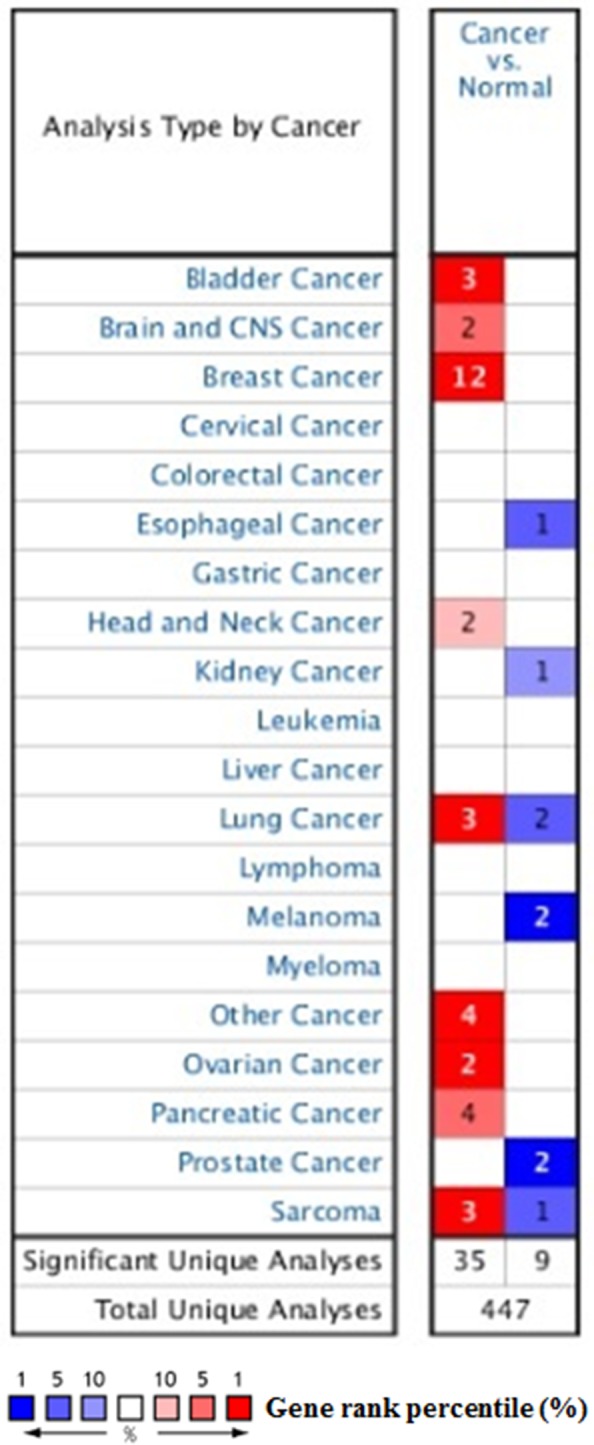
The transcription levels of SDC1 in different types of cancers This graphic was generated from Oncomine, indicating the numbers of datasets with statistically significant (p<0.01) mRNA over-expression (Red) or down-expression (Blue) of SDC1 (different types of cancer vs. corresponding normal tissue). The threshold was designed with following parameters: p-value of 1E-4, fold change of 2, and gene ranking of 10%.

**Figure 2:**
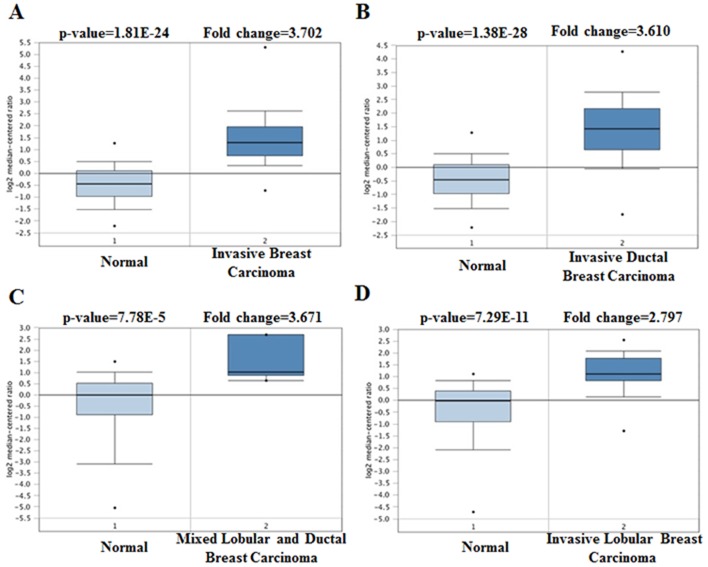
SDC1 analysis in breast cancer (Oncomine database) The box plot comparing specific SDC1 expression in normal (left plot) and cancer tissue (right plot) was derived from Oncomine database. The analysis was shown in invasive breast carcinoma, invasive ductal breast carcinoma, mixed lobular and ductal breast carcinoma, and invasive lobular breast carcinoma relative to normal breast.

**Figure 3:**
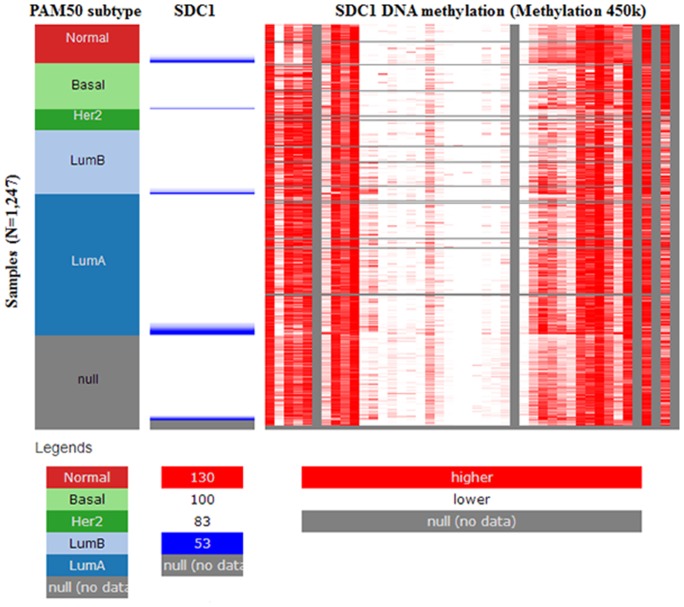
The heat map of SDC1 expression and its DNA methylation status

As a cell surface heparin sulfate proteoglycan (HSPG), the expression of profile of SDC1 was analyzed though using Oncomine database. SDC1 has been identified in human cancers, including hematological malignancies and solid tumors (Figure 1). Oncomine analysis of tumor versus. normal samples revealed that SDC1 mRNA was significantly elevated in invasive breast carcinoma, invasive ductal breast carcinoma, mixed lobular and ductal breast carcinoma, invasive lobular breast carcinoma (Table 1, Figure 2) [19–26]. Through comparing SDC1 expression heat map and its DNA methylation status, we observed that its expression might be negatively related with some CpG sites (blank frame) (Figure 3)

**Table 1:** The significant changes of SDC1 expression in transcription level between different types of breast cancer and normal tissues (*ONCOMINE* database)

Subtype of breast cancer	*p*-value	Fold change	Rank (%)	Sample	Reference
Ductal Breast Carcinoma	7.15E-7	2.567	1	69	[29]
Ductal Breast Carcinoma	3.62E-6	2.317	2	39	[30]
Ductal Breast Carcinoma	1.17E-5	2.754	2	94	[31]
Invasive Ductal Breast Carcinoma	4.86E-5	5.223	1	22	[32]
Invasive Ductal Breast Carcinoma	4.58E-5	3.165	1	23	[33]
Invasive Breast Carcinoma	1.81E-24	3.702	2	137	TCGA
Invasive Ductal Breast Carcinoma	1.38E-28	3.610	4	450	TCGA
Mixed Lobular and Ductal Breast Carcinoma	7.78E-5	3.671	4	68	TCGA
Invasive Lobular Breast Carcinoma	7.29E-11	2.797	5	97	TCGA
Tubular Breast Carcinoma	1.25E-26	3.407	2	211	[34]
Ductal Breast Carcinoma	4.38E-7	2.549	3	47	[35]
Invasive Breast Carcinoma	1.65E-17	2.245	3	59	[36]

### SDC1 mutation in human breast cancer

**Figure 4:**
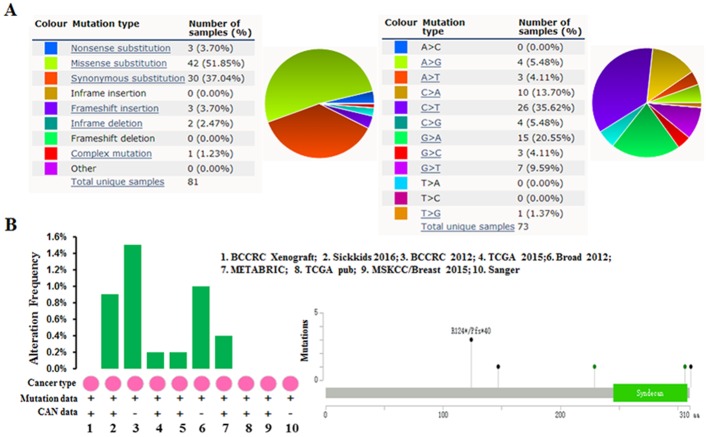
SDC1 mutation in human breast cancer **(A)** Pie-chart showed the percentage of the mutation type of SDC1 in breast cancer according to COSMIC database. **(B)** Alteration frequency of SDC1 mutation in breast cancer was analyzed by using BioPortal.

The pie chart that included the information of mutations of substitution nonsense, missense, synonymous, insertion frame shift, and inframe deletion was generated by performing COSMIC. Substitution missense rate is 51.85% and substitution synonymous rate is 37.04% of mutant samples of breast cancer (Figure 4A). Breast cancer has 35.62% C > T and 20.55% G > A mutation in SDC1 coding strand (Figure 4A). Alteration frequency of SDC1 mutation in breast cancer was identified by performing cBioPortal. Less than 1.6% mutation in the patients with breast cancer was observed (Figure 4B)

### Genetic alterations of SDC1 and clinicopathological parameters in breast cancer patients

**Figure 5:**
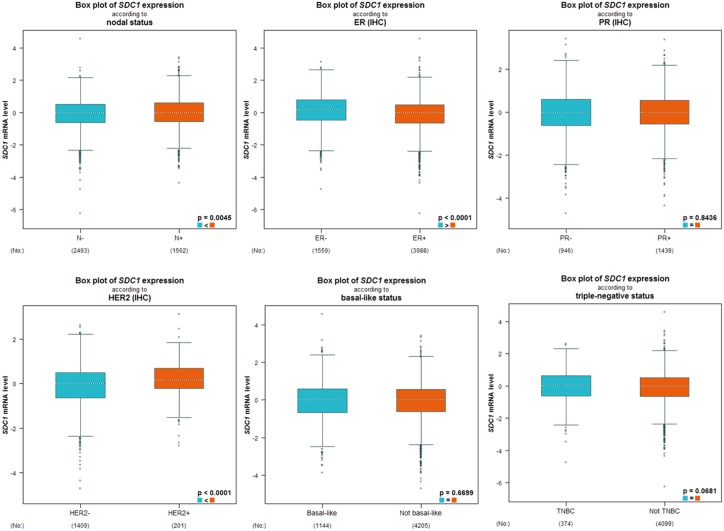
The relationship between mRNA expression of SDC1 and clinicopathological parameters in breast cancer patients Global signifcant different between groups was assessed by Welch’s test to generate *p* value, along with Dunnett-Tukey-Kramer’s.

As displayed in Table 2, we performed a welch’s test to compare the transcription levels of SDC1 between groups of patients by bc-GenExMiner 4.0, basing on different clinicopathological parameters. For age criterion, there was no significant difference between ≤ 51 y and > 51 y groups (Table 2). Breast cancer patients with positive nodal status displayed elevated SDC1 mRNA than negative-nodal patients (Table 2, Figure 5). Estrogen receptor (ER) status was confirmed to be negatively related with SDC1 expression. Conversely, in breast cancer patients with higher epidermal growth factor receptor 2 (HER2), the mRNA levels of SDC1 was significantly increased compared with HER2-negative groups. However, there was no significant difference between positive progesterone receptor (PR) and negative PR groups. Meanwhile, the basal-like and triple-negative breast cancer (TNBC) characteristics also has no difference between positive and negative status (Table 2). In Scarff Bloom & Richardson grade status (SBR) criterion, more advanced SBR grade was associated with the higher mRNA level of SDC1 (Figure 5).

**Table 2:** The relationship between mRNA expression of SDC1 and clinicopathological parameters of breast carcinoma

Variables	SDC1		
	No.^*^	mRNA	*p*-value
**Age**			
≤ 51	1392	-	0.0135
> 51	2210	↑	
**Nodal Statas**			
-	2493	-	0.0045
+	1562	↑	
**ER**			
-	1559	-	<0.0001
+	3988	↓	
**PR**			
-	946	-	0.8436
+	1439	-	
**HER2**			
-	1409	-	<0.0001
+	201	↑	
**Basal-like Status**			
Not	4205	-	0.6699
Basal-like	1144	-	
**Triple-negative Status**			
Not	4099	-	0.0681
TNBC	374	-	

### Association of SDC1 expression and prognosis in breast cancer patients

**Figure 6:**
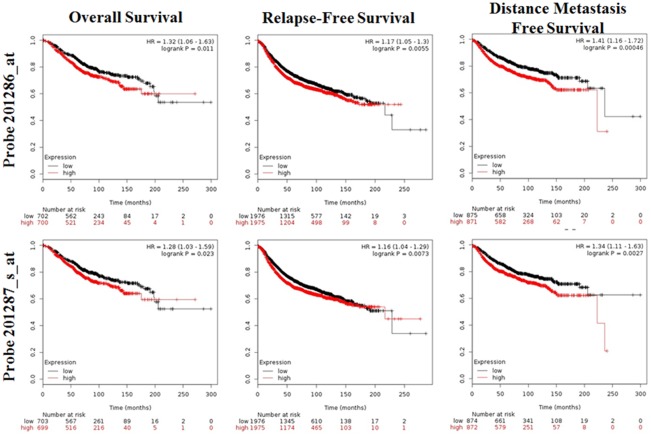
The prognostic value of mRNA level of SDC1 in breast cancer patients (OS, RFS, DMFS in Kaplan-Meier plotter)

**Figure 7:**
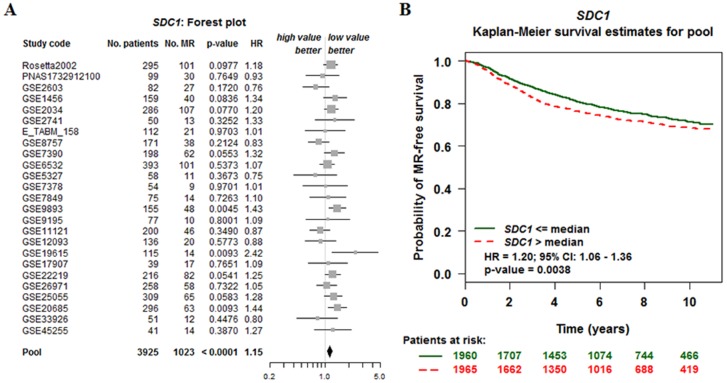
The relationship between SDC1 expression and prognosis metastatic relapse-free survival (MRFS) in breast cancer (bc-GenExMiner v4. 0) **(A)** Forest plots displaying univariate Cox’s analysis of SDC1 expression and the risk of metastatic relapse (MR). **(B)** Kaplan–Meier survival analysis showing the relationship between SDC1 expression and MR-free survival.

Kaplan-Meir analysis revealed that high SDC1 mRNA was correlated with a lower overall survival (OS), relapse-free survival (RFS), distance metastasis free survival (DMFS) in breast cancer (Figure 6). By performing data mining in bc-GenExMiner, we pooled previous available annotated genomic data to analysis the relationship between SDC1 expression and prognosis metastatic relapse-free survival (MRFS) in breast cancer. High CD24 expression was associated with higher risk of metastatic relapse (HR =1.15, 95% CI 1.08 - 1.23, p < 0.0001) (Figure 7A). Similarly, high SDC1 expression was also associated with worse MRFS (HR =1.20, 95% CI 1.06–1.36, p =0.0038) (Figure 7B).

### Co-expression of SDC1 mRNA

**Figure 8:**
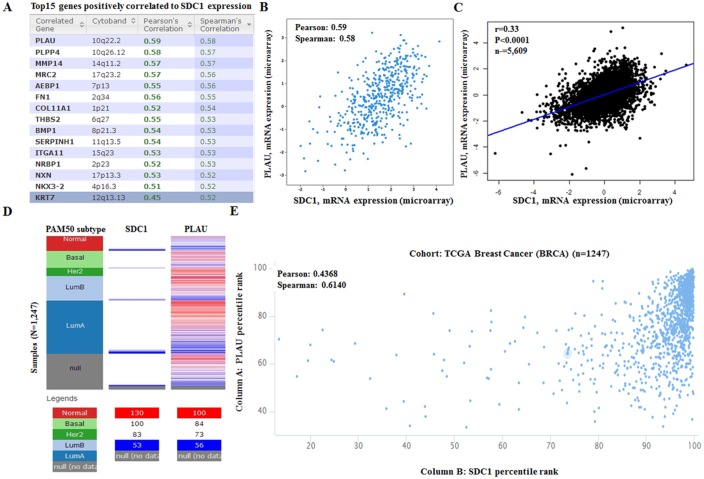
SDC1 mRNA expression is correlated to PLAU mRNA expression in breast cancer **(A)** Top 20 genes positively correlated with SDC1 mRNA expression based on 482 breast cancer patients in TCGA. Analysis was performed using cBioportal (http://cbioportal.org). **(B)** Regression analysis showed that SDC1 and PLAU had high correlation coefficients. **(C)** Data mining in bc-GenExMiner 4.0 confirmed the positive correlation between SDC1 and PLAU mRNA. **(D)** The heat map of SDC1 mRNA and PLAU mRNA expression across PAM50 breast cancer subtypes in TCGA database. Data was analyzed using UCSC Xena (http://xena.ucsc.edu/). **(E)** The correlation between SOX10 and NES mRNA expression in TCGA database.

To further explore the potential regulation of SDC1 in breast cancer, we performed data mining in breast cancer cohort using cBioPortal. PLAU (urokinase plasminogen activator gene, uPA gene) is a top correlated gene (Figure 8A), which can enhance multiple oncogene expression in breast cancer [27, 28]. Regression analysis showed that SDC1 and PLAU had high correlation coefficients (Pearson’s correlation = 0.59; Spearman’s correlation = 0.58) (Figure 8B). Data mining in bc-GenExMiner 4.0 confirmed the positive correlation between SDC1 and PLAU mRNA (Figure 8C). By analyzing breast cancer patient data in TCGA database using UCSC Xena (http://xena.ucsc.edu/), we also confirmed the positive correlation (Figure 8D-8E). These findings suggest that SDC1 might be closely related to the PLAU signaling pathways in breast cancer.

## DISCUSSION

SDC1 is a heparin cell surface proteoglycan acting as a co-receptor for growth factors and chemokines, which strongly associated with the tumor aggressiveness and clinical outcomes [5, 8, 29, 30]. Previous studies have revealed that SDC1 has prognostic value in various types of cancers such as hepatocellular cancer [31], gastric cancer [32], lung cancer [33], and colorectal cancer [7]. In particular, higher expression of SDC1 has been correlated with poorer prognosis for breast cancer patients and correlated with more malignant and higher grade breast cancer tissues [34]. Meanwhile, knockdown of SDC1 expression can inhibits glioma proliferation and invasion through deregulating the c-src/FAK-associated signaling pathway [5]. However, the distinct role of SDC1 expression in the development and metastasis of breast cancer is still unclear.

In the present study, we analyzed the data based on amounts of gene expression with clearly defined parameters between cancer and normal tissues. Oncomine analysis of cancer vs. normal tissue revealed that SDC1 mRNA was significantly over-expressed in invasive breast carcinoma, invasive ductal breast carcinoma, mixed lobular and ductal breast carcinoma, invasive lobular breast carcinoma. Meanwhile, high SDC1 expression is associated with increased risked of age, nodal, HER2 and higher SBR grade status, which predicted fast-growing, spreading tumors. We further analyzed the mutations and alteration frequency of SDC1 using COSMIC and cBioPortal database. Our analysis suggested that the major proportion of mutation in breast cancer is synonymous mutations. Meanwhile, the low alteration frequency was observed in breast cancer. Then, we tried to investigate the mechanisms of SDC1 dysregulation. Through examining its DNA methylation status in TCGA-BRCA, we observed a negative correlation between methylation status of some CpG sites and SDC1 expression. These findings suggest that DNA methylation might be an important mechanism of SDC1 dysregulation in breast cancer.

Survival analysis indicated SDC1 high expression was significantly associated with poor OS, RFS and DMFS. In addition, the following data mining and meta-analysis suggested that high SDC1 expression also associated with increased risk of MR and indicates significantly worse MR-free survival among patients with breast cancer. Depend on these findings, we demonstrated that the expression level of SDC1 might be a useful marker of the prognosis of breast cancer.

Through co-expression and correlation analysis, we observed that PLAU was positively correlated with the expression of SDC1. In fact, as a component of complement and coagulation cascades, PLAU (uPA) can also play a variety of functions in a non-proteolytic fashion, such as cellular adhesion, differentiation, proliferation and migration, via signals triggered by its receptor PLAUR (uPAR) [35, 36]. Dawei Li et al. found that aberrant FOXM1-PLAUR signaling plays an critical role in progression and metastasis of colon cancer [35]. Furthermore, Overexpression of PLAU can largely rescue self-renewal, migration, invasion and vascular formation defects of repressed glioma cells [36]. These results indicated that SDC1 expression might regulated tumor migration and invasion through promoting PLAU expression.

## MATERIALS AND METHODS

### Oncomine database analysis

Oncomine gene expression array datasets (https://www. oncomine.org/resource/login.html), a publicly accessible online cancer microarray database, was performed to determine the mRNA levels of distinct SDC1 in breast cancer [37]. In this study, we compared the clinical specimens of cancer vs. normal control datasets, using the Student’s t–test to generate a *p*-value. The fold change was defined as 2 and p value was set up at 1E-4, whereas the data type was restricted to mRNA.

### COSMIC analysis for SDC1 mutations

The Catalog of Somatic Mutations in Cancer (COSMIC) database (http://www.sange r.ac.uk/cosmic/), the world’s largest and most comprehensive resource for exploring the impact of somatic mutations in human cancer, was performed to analysis the mutations of SDC1. Pie charts were generated for a distribution overview and substitutions on the coding strand in breast cacner.

### UCSC cancer genomics browser analysis

The heat map of SDC1 expression and methylation status and the correlation between SDC1 and PLAU expression were identified using data in TCGA breast cancer cohort (TCGA-BRCA) by performing the UCSC (University of California at Santa Cruz) Cancer Genomics Browser (http://xena.ucsc.edu/) [38–40].

### cBioPortal database analysis

The cBioPortal for Cancer genomics (http://www.cbioportal.org/) is affiliated with Memorial Sloan Kettering Cancer Center and provides integrative analysis of complex cancer genomics and clinical profiles from 105 cancer studies in TCGA pipeline [41, 42]. The term “SDC1” was searched in cBioPortal database and a cross-cancer summary was obtained for it. The search parameters included alterations (amplification, deep deletion, missense mutations), copy-number variance (CNV) from GISTIC and RNA seq data with the default setting. In addition, cBioPortal were performed to identified the positive correlative genes with SDC1 expression in breast cancer.

### Breast cancer gene-expression miner v4.0

Breast Cancer Gene-Expression Miner v4.0 (bc-GenExMiner v4.0), a statistical mining tool of published annotated genomic data including 36 annotated genomic datasets and 5861 breast cancer patients, was used to assess the expression of SDC1 in breast cancer, as well as the association between SDC1 mRNA expression and prognosis in breast cancer patients [43, 44]. Furthermore, the correlation of SDC1 and PLAU were assessed by correlation module of bc-GenExMiner v4.0.

### Kaplan-Meier plotter database analysis

Kaplan-Meier Plotter (http://kmplot.com/analysis/), an online database including gene expression data and clinical data, was performed to analysis the prognostic significance of the mRNA expression of SDC1 in breast cancer [45, 46]. Log rank *p*-value and hazard ratio (HR) with 95% confidence intervals (CI) were calculated and displayed on the webpage.

## CONCLUSIONS

In summary, overexpressed SDC1 was identified in breast cancer than in matched normal tissues and is associated with methylation status of SDC1 promoter. Additionally, SDC1 is positively associated with PLAU and might act as a potential prognostic indicator for breast cancer. However, future research is required to validate our findings and thus promote the clinical utility of SDC1 in breast cancer prognosis evaluation.
